# Rapid Production of Platelet-activating Factor Is Induced by Protein Kinase Cα-mediated Phosphorylation of Lysophosphatidylcholine Acyltransferase 2 Protein*

**DOI:** 10.1074/jbc.M114.558874

**Published:** 2014-04-17

**Authors:** Ryo Morimoto, Hideo Shindou, Megumi Tarui, Takao Shimizu

**Affiliations:** From the ‡ Department of Lipid Signaling, Research Institute, National Center for Global Health and Medicine, 1-21-1 Toyama, Shinjuku-ku, Tokyo 162-8655, Japan,; the § Department of Biochemistry and Molecular Biology, Faculty of Medicine, The University of Tokyo, 7-3-1 Hongo, Bunkyo-ku, Tokyo 113-0033, Japan, and; the ¶ Core Research for Evolutional Science and Technology (CREST), Japan Science and Technology Agency, 4-1-8 Honcho, Kawaguchi, Saitama 332-0012, Japan

**Keywords:** Enzyme Catalysis, Inflammation, Macrophages, Membrane Lipids, Phosphorylation, Protein Kinase C (PKC), Platelet-activating Factor, Lysophospholipid Acyltransferase

## Abstract

Platelet-activating factor (PAF), a potent proinflammatory lipid mediator, is synthesized rapidly in response to extracellular stimuli by the activation of acetyl-CoA:lyso-PAF acetyltransferase (lyso-PAFAT). We have reported previously that lyso-PAFAT activity is enhanced in three distinct ways in mouse macrophages: rapid activation (30 s) after PAF stimulation and minutes to hours after LPS stimulation. Lysophosphatidylcholine acyltransferase 2 (LPCAT2) was later identified as a Ca^2+^-dependent lyso-PAFAT. However, the mechanism of rapid lyso-PAFAT activation within 30 s has not been elucidated. Here we show a new signaling pathway for rapid biosynthesis of PAF that is mediated by phosphorylation of LPCAT2 at Ser-34. Stimulation by either PAF or ATP resulted in PKCα-mediated phosphorylation of LPCAT2 to enhance lyso-PAFAT activity and rapid PAF production. Biochemical analyses showed that the phosphorylation of Ser-34 resulted in augmentation of *V*_max_ with minimal *K_m_* change. Our results offer an answer for the previously unknown mechanism of rapid PAF production.

## Teaser

**Background:** The mechanism for rapid biosynthesis of platelet-activating factor (PAF), a potent proinflammatory lipid mediator, is unclear.

**Results:** Phosphorylation of lysophosphatidylcholine acyltransferase 2 (LPCAT2) at Ser-34 via PKCα enhanced rapid PAF biosynthesis following PAF or ATP stimulation.

**Conclusion:** The molecular basis for rapid PAF biosynthesis in response to inflammatory stimuli is elucidated.

**Significance:** These data suggest a better understanding of PAF production in early-phase inflammation.

## Introduction

Platelet-activating factor (PAF,^3^ 1-*O*-alkyl-2-acetyl-*sn*-glycero-3-phosphocholine) is a potent proinflammatory lipid mediator that triggers various cellular functions through binding to its G protein-coupled receptor, PAF receptor (PAFR), resulting in pleiotropic biological effects such as platelet activation, airway constriction, hypotension, and systemic anaphylaxis (1–5). Production of lipid mediators is an important step for regulating inflammation and immune responses. The most important feature of lipid mediators is that they are synthesized rapidly from precursor biomembrane phospholipids, adapting to the environment around cells. Specific enzymes synthesize most lipid mediators on demand in a Ca^2+^-dependent manner. In response to extracellular stimuli, PAF is synthesized rapidly in the remodeling pathway from its precursor membrane phospholipid, 1-*O*-alkyl-2-acyl-*sn*-glycero-3-phosphocholine (1-alkyl-PC). Under inflammatory conditions, 1-alkyl-PC is hydrolyzed in a Ca^2+^-dependent manner by the action of cytosolic phospholipase A_2_ to produce polyunsaturated fatty acid and lyso-PAF, a lysophosphatidylcholine (LPC) derivative with an alkyl ether bond at the *sn*-1 position. Biosynthetic enzymes of various lipid mediators subsequently utilize polyunsaturated fatty acid and lyso-PAF as substrates. Our previous studies showed that macrophages and neutrophils from cytosolic phospholipase A_2_α-deficient mice are unable to produce PAF in response to extracellular stimuli (6, 7). PAF is synthesized from lyso-PAF by acetyl-CoA:lyso-PAF acetyltransferase (lyso-PAFAT (EC: 2.3.1.67)) (2). Endogenous lyso-PAFAT activity has been reported initially in 1980 and partially characterized (8). Our laboratory identified a molecular entity of lyso-PAFAT, lysophosphatidylcholine acyltransferase 2 (LPCAT2) (9). This enzyme is a 60-kDa protein with three putative transmembrane domains and several EF-hand motifs and is localized in endoplasmic reticulum (1). Although the biosynthesis of PAF is understood poorly, its degradation has been well characterized. Deacetylation of PAF is catalyzed by PAF acetyl hydrolase and leads to the loss of its activity (10–12).

LPCAT2 also possesses lysophosphatidylcholine acyltransferase (LPCAT (EC: 2.3.1.23)) activity that catalyzes the incorporation of arachidonoyl-CoA into membrane phosphatidylcholine (PC). In fact, 1-alkyl-PC represents approximately half of the phospholipid content in the biomembranes of hematopoietic cells (13). Thus, LPCAT2 functions in the biosynthetic pathway of phospholipids known as the Lands cycle or remodeling pathway, which is responsible for generating the diversity of phospholipid species in biomembranes (14). LPCAT2 also influences the fatty acid content of cellular membranes and the availability of substrates for cellular lipid mediator production.

There are a number of reports showing that lyso-PAFAT activity is enhanced by several distinct mechanisms. We reported previously that, in mouse macrophages, lyso-PAFAT activity was increased within 30 s after PAFR stimulation (15) and within 30 min or 16 h after LPS stimulation (9, 16). LPS stimulation for 16 h increased mRNA of LPCAT2 (9). LPS stimulation for 30 min activated LPCAT2 by Ser-34 phosphorylation via the p38 MAPK/MAPK-activated protein kinase 2 (MK2)-dependent pathway (16). However, the mechanism of lyso-PAFAT activation by PAF stimulation on the order of seconds was unclear. Several studies have reported that activation of endogenous lyso-PAFAT activity is dependent on protein kinase A, PKC, or p38 MAPK pathways (17–19), but the regulatory mechanisms and the gene responsible for the rapid lyso-PAFAT activity in response to PAF have not been clarified.

In this study, we identified phosphorylation at Ser-34 of LPCAT2 via PKCα signaling as the underlying mechanism that led to rapid enhancement of lyso-PAFAT activity following PAF- or ATP-stimulation. Enzymological analyses showed that phosphorylation increased the *V*_max_ value of LPCAT2, whereas the *K_m_* value was almost unchanged. Our report identifies the enzyme and signaling pathway that mediate rapid activation of PAF production, which is critical in the early stages of inflammatory responses.

## EXPERIMENTAL PROCEDURES

### 

#### 

##### Materials

PC from frozen egg yolk, LPS from *Salmonella minnesota*, ATP, anti-FLAG M2 antibody, and anti-β-actin antibody were from Sigma. Deuterium-labeled d_4_-16:0 lyso-PAF, d_4_-16:0 PAF, 16:0 lyso-PAF, and 16:0 methylcarbamyl PAF (mcPAF, a non-hydrolyzed analog of PAF (20)) were from Cayman Chemical Co. (Ann Arbor, MI). Arachidonoyl-CoA was from Avanti Polar Lipids (Alabaster, AL). [^3^H]Acetyl-CoA (129.5 GBq/mmol) and horseradish peroxidase-linked anti-rabbit or anti-mouse IgGs were from GE Healthcare (Buckinghamshire, UK). [1-^14^C]Arachidonoyl-CoA (2.22 GBq/mmol) was from Moravec Biochemicals (Brea, CA). TLC silica gel plates (type 5721) were from Merck (Darmstadt, Germany). Acetyl-CoA, dimethyl sulfoxide, acrylamide, and Phos tag acrylamide were from Wako (Osaka, Japan). U73122 (PLCβ inhibitor), U73343 (an inactive analog of U73122), bisindolylmaleimide I (BIM-I, a PKC inhibitor), Ro-31-8220, Ro-31-8425, bisindolylmaleimide V (BIM-V, an inactive analog of BIM-I), and 12-*O*-tetradecanoylphorbol-13-acetate (TPA) were from Calbiochem. The siRNAs (ON-TARGETplus Non-targeting Pool D-001810-10−20, ON-TARGETplus SMARTpool L-040348-00-0005 (PKCα), and L-052470-01-0005 (LPCAT2)) were from Thermo Scientific (Waltham, MA). The cell line Nucleofector kit V was from Lonza (Basel, Switzerland). Anti-MK2, anti-phospho-Thr-334-MK2, and anti-PKCα antibodies were from Cell Signaling Technology (Beverly, MA). The protease inhibitor mixture, EDTA-free Complete, was from Roche. The anti-LPCAT2 and anti-phospho-LPCAT2 antibodies and FLAG-LPCAT1, FLAG-LPCAT2, S34A, and S34D plasmids have been described previously (16).

##### Mice

C57BL/6J mice were obtained from Clea Japan, Inc. (Tokyo, Japan). Mice were maintained in a light-dark cycle with lights on from 08:00–20:00 h at 22 °C. Mice were fed with a standard laboratory diet and water *ad libitum*. All animal studies were conducted in accordance with the guidelines for animal research at The University of Tokyo and were approved by The University of Tokyo Ethics Committee for Animal Experiments.

##### Isolation of Mouse Peritoneal Macrophages

Thioglycolate-induced mouse peritoneal macrophages were isolated as described previously (15). Cells were cultured for 16 h before inhibitor treatment or mcPAF stimulation.

##### Preparation of Cell Lysates

Cells were pretreated with the following reagents for the indicated times: 10 μm BAPTA/AM, 10 μm U73122, or 10 μm U73343 for 10 min; 1–20 μm of BIM-I, 1–10 μm of Ro-31-8220, 1–10 μm of Ro-31-8425, and 10 μm of BIM-V for 30–60 min; and 100 nm TPA for 24 h. Cells were then stimulated with 200 nm mcPAF or 250 μm ATP for 30 s. After stimulation, cells (peritoneal macrophages, CHO-S cells stably expressing PAFR (21), non-transfected RAW264.7 cells, and RAW264.7 cells stably expressing the vector or FLAG-LPCAT2 (16)) were washed with ice-cold buffer containing 20 mm Tris-HCl (pH 7.4), 0.3 m sucrose, and 1 mm sodium orthovanadate and collected in buffer containing 20 mm Tris-HCl (pH 7.4), 1 mm sodium orthovanadate, 5 mm 2-mercaptoethanol, and 1× EDTA-free complete protease inhibitor mixture. Subsequently, cells were sonicated twice on ice for 30 s each time and centrifuged at 9000 × *g* for 10 min at 4 °C to remove cellular debris, intact cells, and mitochondria.

For peritoneal macrophages, the resultant supernatant from the centrifugation at 9000 × *g* was separated at 100,000 × *g* for 1 h at 4 °C. The resultant pellet was resuspended with ice-cold buffer containing 20 mm Tris-HCl (pH 7.4), 1 mm sodium orthovanadate, 5 mm 2-mercaptoethanol, and 1× EDTA-free Complete protease inhibitor mixture. The concentration of each protein was measured by the Bradford method (22) using protein assay solution (Bio-Rad). Bovine serum albumin (fraction V, fatty acid-free, Sigma) served as a standard.

##### Transfection of CHO-S Cells Stably Expressing PAFR

CHO-S cells stably expressing PAFR (CHO-S-PAFR cells) (21) were seeded in 10-cm collagen-coated dishes 24 h before transfection. Empty vector, FLAG-LPCAT1, FLAG-LPCAT2, S34A, and S34D plasmids (15 μg of each) were transfected into CHO-S-PAFR cells using 30 μl of Lipofectamine 2000 (catalog no. LF2000, Invitrogen). Twenty-four hours after transfection, cells from each 10-cm dish were divided into three 6-cm dishes and incubated at 37 °C in 5% CO_2_ for 2 h. The medium was then changed to DMEM containing 0.5% BSA. Cells were cultured without serum for 24 h and then stimulated with 200 nm mcPAF for 30 s.

For the knockdown of PKCα, CHO-S-PAFR cells were seeded in 6-cm collagen-coated dishes 24 h before transfection of 180 pmol siRNA using 17 μl of RNAi MAX (Invitrogen). The cells were incubated for 24 h and subsequently cotransfected with 4 μg of FLAG-LPCAT2. After another 24 h of incubation, cells from each 6-cm dish were divided into two 6-cm dishes and incubated for 2 h. The medium was changed to DMEM containing 0.5% BSA. The cells were cultured without serum for 24 h and then stimulated with 200 nm mcPAF for 30 s.

##### Transfection of RAW264.7 Cells Stably Expressing FLAG-LPCAT2

RAW264.7 cells stably expressing FLAG-LPCAT2 were suspended in 100 μl of Nucleofector kit V and mixed with 30 pmol PKCα siRNA or negative control siRNA. The mixture in the cuvette was set onto the Amaxa Nucleofector and electroporated with program D-032. Then, cells were seeded onto 6-cm dishes. Forty-eight hours after transfection, cells were stimulated with 250 μm ATP for 30 s.

##### Western Blot Analysis

Western blot analyses were performed as described previously (23). To detect band shifts that represent phosphorylated proteins, SDS-PAGE gels containing 50 μm Phos tag acrylamide and 100 μm manganese ions (Mn^2+^) were used. The Phos tag forms a complex with two Mn^2+^ ions and serves as a phosphate-binding molecule (24). The complex is used for phosphate affinity SDS-PAGE, which results in the upward mobility shift of phosphorylated proteins. Reacted proteins were detected using the LAS4000 or LAS500 imaging systems (GE Healthcare).

##### Assay of Lyso-PAFAT and LPCAT

The lyso-PAFAT and LPCAT assays were performed using radioisotopes, as described previously (9, 25), or LC-MS/MS. The *K_m_* and *V*_max_ of LPCAT2 and phospho-LPCAT2 activities were measured under the following conditions: 5 μg/ml microsomal proteins (pellet after centrifugation at 100,000 × *g* for 1 h) were incubated in buffer containing 100 mm Tris-HCl (pH 7.4), 0.015% Tween 20, 1 μm CaCl_2_, deuterium-labeled lyso-alkyl-PC (d_4_-16:0 lyso-PAF), and acetyl-CoA or arachidonoyl-CoA at 37 °C for 5 min. The concentrations of each substrate are described in Fig. 3, *D–G*. Other assays were performed under the following conditions: 10 μg/ml proteins (supernatant after centrifugation at 9000 × *g* for 10 min) were incubated in buffer containing 100 mm Tris-HCl (pH 7.4), 0.015% Tween 20, 1 μm CaCl_2_, 5 μm d_4_-16:0 lyso-PAF and 1 mm acetyl-CoA at 37 °C for 5 min. Lipids were extracted with methanol containing dimyristoyl phosphatidylcholine (14:0/14:0 PC) or 17:0 LPC as an internal standard and subsequently analyzed by LC-MS/MS.

##### Extraction and Purification of Cellular PAF Product

For endogenous PAF analysis, lipids from cells grown on 35-mm dishes were extracted with 500 μl of methanol containing d4–16:0 PAF as an internal standard and purified with Oasis HLB cartridges (Waters, Milford, MA) as described previously (26, 27).

##### Analysis of Enzyme Assay Products and Endogenous PAF Production by LC-MS/MS

Chromatography was performed on an Acquity^TM^ UPLC BEH C_8_ column (1.7 μm, 2.1 × 30 mm, Waters) using an Acquity^TM^ ultra-performance LC system (Waters) as described previously (28). Lipids were measured by a TSQ Vantage Triple Stage Quadrupole mass spectrometer (Thermo Scientific) equipped with a HESI-II electrospray ionization source, as described previously (29). Endogenous PAF from peritoneal macrophages and RAW264.7 cells was measured using LC-MS/MS. Chromatography was carried out on a Kinetex C_8_ column (2.1 × 150 mm, Phenomenex, Torrance, CA). Nexera UHPLC (Shimadzu, Kyoto, Japan) was connected to an LCMS-8040 (Shimadzu) mass spectrometer (25). Deuterium-labeled d4–16:0 PAF and non-labeled PAF were monitored by selected reaction monitoring transition (*m/z* 528.4–184.1 and 524.4–184.1, respectively) in positive ion scan mode. Deuterium-labeled 1-alkyl-PC was monitored by selected reaction monitoring transition (*m/z* 832.6–303.2) in negative ion scan mode. Signal intensities were determined relative to the internal standard or the percentage of the positive control. Mass spectra were processed using Xcalibur 2.0 software (Thermo Scientific) or LabSolutions 5.53 software (Shimadzu).

##### Quantitative Real-time PCR

Total RNAs were prepared using the RNeasy mini kit (Qiagen), and first strand cDNA was subsequently synthesized using Superscript III (Invitrogen). The PCRs were performed using Fast SYBR Green Master Mix and the StepOnePlus^TM^ real-time PCR system (Applied Biosystems). The following primers were used: *Lpcat2*, a 64-bp fragment, CCCTGCCAATACAGAAGAGATCA (forward) and GCCGTCCTCATCAACATCAA (reverse); *Prkca*, a 172-bp fragment, GTGTCCTACCCCAAATCCTTGTC (forward) and GTTGGATCTCCCTGTTCTCCAGT (reverse); and *Rplp0*, a 136-bp fragment, CTGAGATTCGGGATATGCTGTTG (forward) and AAAGCCTGGAAGAAGGAGGTCTT (reverse).

##### Software

All statistical calculations were performed using Prism 5 (GraphPad Software, La Jolla, CA).

## RESULTS

### 

#### 

##### Activation and Phosphorylation of LPCAT2 Following mcPAF Stimulation

**FIGURE 1.**
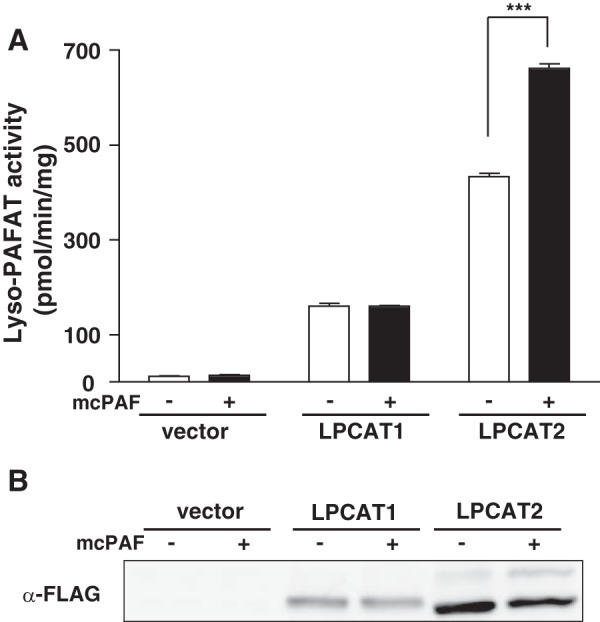
**LPCAT2 activation and phosphorylation.** CHO-S-PAFR cells transfected with FLAG-LPCAT1, FLAG-LPCAT2, or empty vector were stimulated with 200 nm mcPAF for 30 s. *A*, mcPAF enhanced the activity of LPCAT2 but not LPCAT1. Results are expressed as the mean + S.D. of an experiment performed in triplicate. Three independent experiments showed similar results. *B*, the phosphorylation of LPCAT1 and LPCAT2 was examined by Phos tag acrylamide gel electrophoresis and Western blot analysis. LPCAT2 showed an upward shifted band indicative of phosphorylation, which was increased after mcPAF stimulation. ***, *p* < 0.001; analysis of variance and Tukey's multiple comparison test.

There are two known enzymes that possess lyso-PAFAT activity *in vitro*: LPCAT1 and LPCAT2 (9, 25). In inflammatory cells, LPCAT2 is the predominant lyso-PAFAT to produce PAF in response to several stimuli (9). To examine the different characteristics of the two lyso-PAFATs, FLAG-tagged LPCAT1 and LPCAT2 were transiently transfected into CHO-S cells stably expressing PAFR (CHO-S-PAFR cells). CHO-S-PAFR cells can respond to PAF by several signaling events, including Ca^2+^ response (21). Cells were stimulated with mcPAF for 30 s, and lyso-PAFAT activities in the 9000 × *g* centrifugation supernatants were examined by incorporation of [^3^H]-labeled acetyl-CoA into PAF. Although the activity of LPCAT1 was unchanged after mcPAF stimulation, that of LPCAT2 was enhanced (Fig. 1*A*). Lyso-PAFAT activity was not detected in vector-transfected samples.

Previously, we observed that phosphorylation of LPCAT2 at a single residue, Ser-34, occurs 30 min after LPS stimulation and leads to an increase of lyso-PAFAT activity (16). To investigate the mechanism of rapid LPCAT2 activation, we examined the phosphorylation status of LPCAT2 after 30-s exposure to mcPAF by Phos tag acrylamide gel electrophoresis and Western blot analysis. An upward shifted band indicative of phosphorylation was detected for FLAG-LPCAT2 but not FLAG-LPCAT1, indicating that LPCAT2 is rapidly phosphorylated after mcPAF stimulation to enhance its enzymatic activity (Fig. 1*B*).

##### Different Pathways Mediate LPCAT2 Phosphorylation Following mcPAF and LPS Stimulation

**FIGURE 2.**
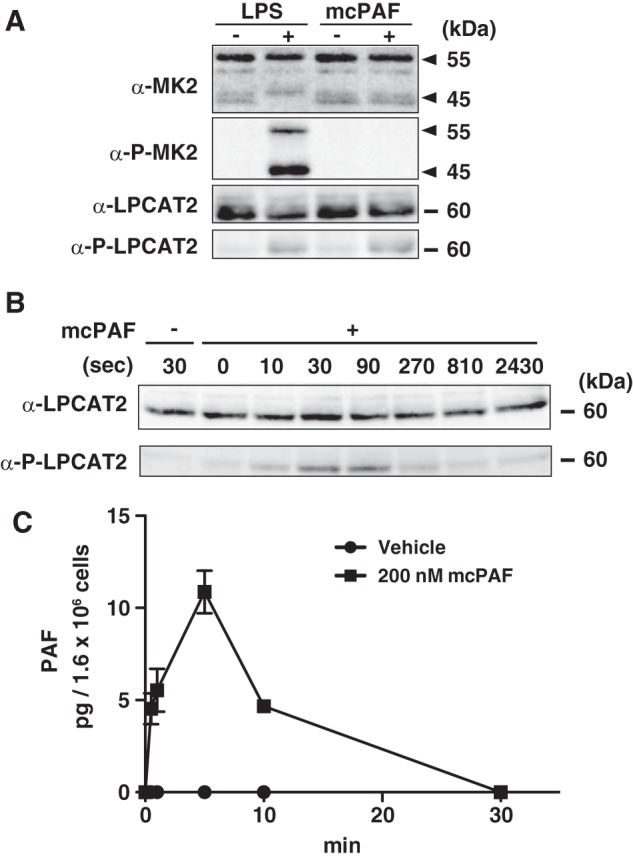
**Signaling pathway for LPCAT2 phosphorylation.** Peritoneal macrophages were stimulated with 100 ng/ml LPS for 30 min or 200 nm mcPAF for 30 s. *A*, phosphorylation of MK2 and LPCAT2 was detected by Western blot analysis. Phosphorylation of LPCAT2 at Ser-34 occurred after stimulation with either compound. Phospho-MK2 reactive bands were detected following LPS but not mcPAF stimulation. *Arrowheads* indicate MK2 splice variants. *B*, the time course of LPCAT2 phosphorylation following mcPAF stimulation was determined by Western blot analysis. Phosphorylation of LPCAT2 at Ser-34 reached a maximum within 30 s. Three independent experiments were performed with similar results (*A* and *B*). *C*, PAF production from peritoneal macrophages was measured by LC-MS/MS. Lipids were extracted with methanol containing deuterium-labeled PAF as an internal standard. Results are expressed as the mean + S.E. of three experiments.

To determine the phosphorylation site of LPCAT2 following mcPAF stimulation, peritoneal macrophages were stimulated with 100 ng/ml LPS for 30 min or 200 nm mcPAF for 30 s, and the microsomal proteins (100,000 × *g* pellet) were analyzed by Western blot analysis using antibodies to detect total or phospho-Ser-34 LPCAT2. Phosphorylated LPCAT2 was barely detectable in untreated cells but detected readily following either 30 min of LPS or 30 s of mcPAF stimulation. To examine whether phosphorylation of LPCAT2 following mcPAF stimulation is also MK2-dependent, the samples were probed with antibodies that recognize total or activated MK2. Phosphorylated MK2 was observed following LPS but not mcPAF stimulation (Fig. 2*A*), indicating that the rapid phosphorylation of LPCAT2 in response to mcPAF is independent of MK2 signaling and occurs via a separate signaling pathway.

##### Time Course of LPCAT2 Phosphorylation and PAF Production Following mcPAF Stimulation

To investigate the rapid time course of LPCAT2 phosphorylation, peritoneal macrophages were stimulated with 200 nm mcPAF for varying times (0–2430 s), and microsomal proteins were analyzed by Western blot analysis using antibodies to detect total or phospho-Ser-34 LPCAT2. The amount of total LPCAT2 was similar in all samples. Phosphorylated LPCAT2 was not detected in vehicle-treated cells, but stimulation with mcPAF rapidly induced phosphorylation of LPCAT2 in as little as 10 s, reached a maximum by 30–90 s, and was decreased by 270 s (Fig. 2*B*), consistent with our previous study (15). Next, we examined whether endogenous PAF production correlated with the phosphorylation of LPCAT2 following mcPAF stimulation. A rapid and transient increase in PAF occurred within 30 s and was at a maximum at 5 min but undetectable by 30 min, consistent with its rapid synthesis and degradation (Fig. 2*C*).

##### Ser-34 Phosphorylation Is Required for mcPAF-induced LPCAT2 Activation

**FIGURE 3.**
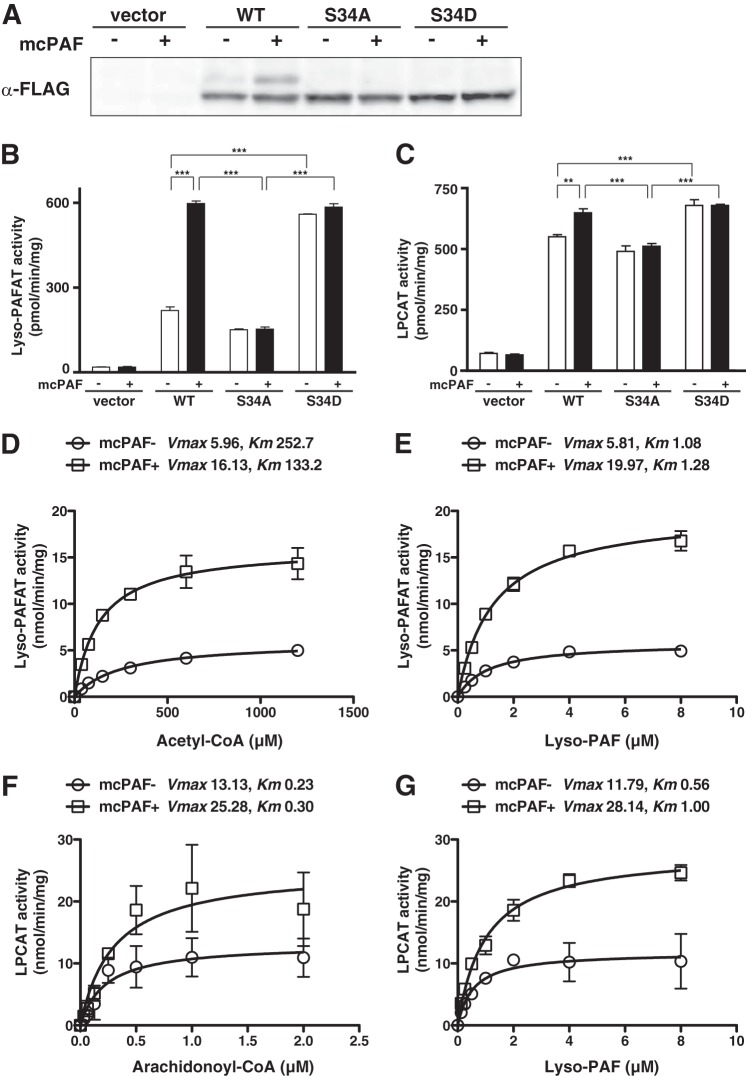
**Site-directed mutagenesis of LPCAT2 and effect of LPCAT2 phosphorylation on enzymatic activities.** CHO-S-PAFR cells transfected with vector, WT, or mutant (S34A and S34D) LPCAT2 were stimulated with 200 nm mcPAF for 30 s. *A*, phosphorylation was examined by Phos tag Western blot analysis. An upward-shifted band was detected in WT but not S34A or S34D mutants following mcPAF stimulation. Lyso-PAFAT (*B*) and LPCAT (*C*) activities were analyzed by measuring the incorporation of radiolabeled acyl-CoA ([^3^H]acetyl-CoA or [^14^C]arachidonoyl-CoA) into PAF or PC, respectively. WT LPCAT2 showed enhanced lyso-PAFAT and LPCAT activities following mcPAF stimulation. The S34A mutant did not show enhanced activities following stimulation, whereas the S34D showed enhanced basal activity compared with the WT but was not increased following mcPAF stimulation. Results are expressed as the mean + S.D. of an experiment performed in triplicate. Four independent experiments were performed with similar results. **, *p* < 0.01; ***, *p* < 0.001; analysis of variance and Tukey's multiple comparison test. Lyso-PAF acetyltransferase assays were performed using 5 μm deuterium-labeled lyso-PAF and the indicated concentrations of acetyl-CoA (*D*) or 1 mm acetyl-CoA and increasing concentrations of lyso-PAF (*E*). LPCAT assays were conducted with 5 μm deuterium-labeled lyso-PAF and the indicated concentrations of arachidonoyl-CoA (*F*) or 2.5 μm arachidonoyl-CoA and increasing concentrations of deuterium-labeled lyso-PAF (*G*). Phosphorylation of LPCAT2 affected the *V*_max_ value of its catalytic activities. The data represent the mean ± S.D. of the difference between LPCAT2- and vector-transfected samples in triplicate measurements. The units of *V*_max_ and *K_m_* are nanomoles/minute/milligram and micromolar, respectively. The results are representative of two independent experiments with similar results.

To further examine the role of Ser-34 phosphorylation following mcPAF-stimulation, two FLAG-tagged mutant constructs (S34A and S34D) of LPCAT2 were transiently expressed in CHO-S-PAFR cells. The cells were stimulated with mcPAF for 30 s, and the cellular proteins were analyzed by Phos tag acrylamide gel electrophoresis and Western blot analysis. An upward mobility shift in LPCAT2 indicative of phosphorylation was detected in WT LPCAT2 following mcPAF-stimulation but not in the S34A or S34D mutant proteins (Fig. 3*A*). Next, we examined the effect of phosphorylation on the dual activities of LPCAT2 (lyso-PAFAT and LPCAT) by measuring the incorporation of radiolabeled acyl-CoA ([^3^H]acetyl-CoA or [^14^C]arachidonoyl-CoA) into PAF or PC, respectively. Both the lyso-PAFAT and LPCAT activities of WT LPCAT2 were enhanced by mcPAF stimulation (Fig. 3, *B* and *C*). Basal S34A activities were similar to WT but not increased by mcPAF stimulation. Conversely, basal activities of LPCAT2 containing the S34D mutation, which functions as a phosphomimetic, were higher than in the WT, but enhancement by mcPAF stimulation was not observed (Fig. 3*B*). Because the expression level of each construct was similar (Fig. 3*A*), our results indicate that both the lyso-PAFAT and LPCAT activities of LPCAT2 are enhanced by Ser-34 phosphorylation in response to mcPAF stimulation.

##### Effect of Ser-34 Phosphorylation on the Enzymatic Activities of LPCAT2

Next, we investigated how Ser-34 phosphorylation affects enzymatic activities of LPCAT2 using microsomal proteins from CHO-S-PAFR cells overexpressing LPCAT2. Proteins from mcPAF-treated cells exhibited higher *V*_max_ values than control proteins in both lyso-PAFAT and LPCAT activities *in vitro* (Fig. 3, *D–G*), potentially because of a conformational change in LPCAT2, which is phosphorylated at Ser-34. Changes in the *K_m_* values were minimal following mcPAF stimulation.

##### Signaling Pathways That Mediate LPCAT2 Phosphorylation following mcPAF Stimulation

Although the phosphorylation of LPCAT2 by 30 min of LPS stimulation was dependent on MK2, mcPAF stimulation for 30 s utilized a different pathway (Fig. 2*A*). Therefore, we examined signaling pathway activation after mcPAF stimulation.

**FIGURE 4.**
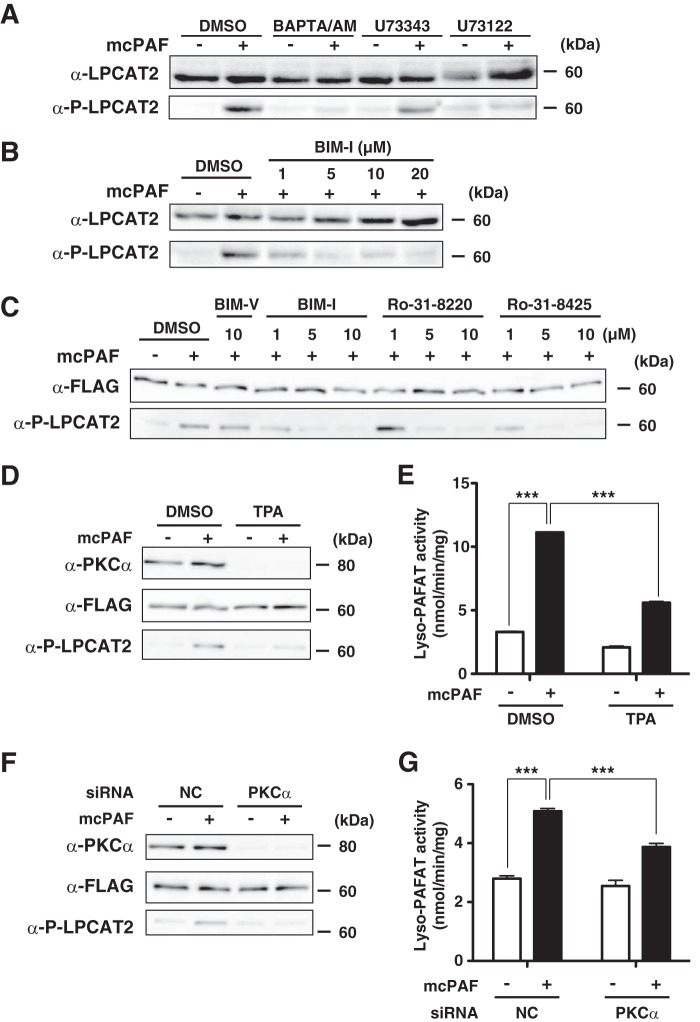
**Conventional PKC-dependent LPCAT2 phosphorylation.** Peritoneal macrophages were preincubated with or without inhibitors and subsequently stimulated with 200 nm mcPAF for 30 s. Microsomal proteins were subjected to Western blot analysis. *A*, pretreatment with 10 μm BAPTA/AM or 10 μm U73122, but not an inactive analog, U73343, abolished LPCAT2 phosphorylation. *DMSO*, dimethyl sulfoxide. *B*, pretreatment with the PKC inhibitor BIM-I inhibited mcPAF-induced LPCAT2 phosphorylation in a dose-dependent manner. 5 μm BIM-I was sufficient to achieve maximum inhibition. *C*, CHO-S-PAFR cells transfected with FLAG-LPCAT2 were preincubated with or without PKC inhibitors for 1 h and stimulated with 200 nm mcPAF for 30 s. Western blot analysis showed that each of the three different PKC inhibitors abolished the phosphorylation in a dose-dependent manner. Pretreatment with BIM-V, an inactive analog of BIM-I, had no effect. *D*, CHO-S-PAFR cells transfected with FLAG-LPCAT2 were pretreated with 100 nm TPA for 24 h and stimulated with mcPAF. TPA decreased PKCα protein and phosphorylation of LPCAT2 by mcPAF. *E*, pretreatment with TPA also resulted in reduced lyso-PAFAT activation following mcPAF stimulation, as measured by incorporation of acetyl-CoA into deuterium-labeled PAF. *F* and *G*, PKCα siRNA and FLAG-LPCAT2 were cotransfected into CHO-S-PAFR cells. Reduction of PKCα protein expression by its siRNA transfection was associated with reduced mcPAF-induced phosphorylation of LPCAT2 and lyso-PAFAT activation following mcPAF stimulation. Results are expressed as the mean + S.D. of an experiment performed in triplicate. *NC*, negative control. ***, *p* < 0.001; analysis of variance and Tukey's multiple comparison test. Three (*A–C*) or four (*D*--*G*) independent experiments were performed with similar results.

It has been shown that, following PAFR stimulation, intracellular Ca^2+^ concentration is increased rapidly via the G_q/11_-PLCβ-inositol 1,4,5-trisphosphate pathway (30–32). We also detected an increased intracellular Ca^2+^ concentration in peritoneal macrophages and CHO-S-PAFR cells within 30 s of mcPAF stimulation (data not shown). Therefore, we examined the effect of pretreatment with a cytosolic Ca^2+^ chelator (BAPTA/AM) (33, 34) as well as a PLCβ inhibitor (U73122) or an inactive analog (U73343) (35) on LPCAT2 phosphorylation. Peritoneal macrophages were pretreated with each compound for 10 min and then stimulated with mcPAF for 30 s. The phosphorylation status of LPCAT2 was analyzed by Western blot analysis. Both inhibitors abolished mcPAF-induced LPCAT2 phosphorylation (Fig. 4*A*), indicating that this phosphorylation requires PLCβ activation and intracellular Ca^2+^ elevation.

Next, we investigated kinase activation following PAFR signaling. Because previous reports showed that PKC is activated rapidly after PAFR stimulation (36, 37), the involvement of PKC in LPCAT2 phosphorylation was examined using the PKC inhibitor BIM-I (38). Peritoneal macrophages were pretreated with various concentrations of BIM-I (1–20 μm) for 30 min and then stimulated with mcPAF for 30 s. Microsomal proteins were analyzed by Western blot analysis using antibodies against total or phospho-LPCAT2. mcPAF-induced LPCAT2 phosphorylation was diminished by BIM-I pretreatment in a dose-dependent manner. Pretreatment with 5 μm BIM-I was sufficient to obtain this effect (Fig. 4*B*).

Other PKC-specific inhibitors (Ro-31-8220 and Ro-31-8425) also showed dose-dependent inhibition of LPCAT2 phosphorylation in CHO-S-PAFR cells transiently expressing FLAG-LPCAT2, whereas pretreatment with BIM-V, an inactive analog of BIM-I, had no effect (Fig. 4*C*). These compounds selectivity inhibit conventional PKC (cPKC) isozymes (39) over the Ca^2+^-independent PKC isozymes (40). We conclude that Ca^2+^ cPKC signaling regulates the phosphorylation of LPCAT2 following mcPAF stimulation.

Next, CHO-S-PAFR cells transiently expressing FLAG-LPCAT2 were pretreated with 100 nm TPA for 24 h. Consistent with previous reports (41, 42), prolonged exposure to TPA caused down-regulation of PKCα protein, whereas expression of LPCAT2 was not affected (Fig. 4*D*). The phosphorylation of LPCAT2 by mcPAF-stimulation was reduced by TPA treatment, accompanied by a decrease in lyso-PAFAT activity *in vitro* (Fig. 4*E*).

To further confirm the involvement of PKCα in phosphorylation of LPCAT2 at Ser-34, PKCα siRNA and FLAG-LPCAT2 were cotransfected into CHO-S-PAFR cells. PKCα protein was reduced by the knockdown, and mcPAF-induced phosphorylation of LPCAT2 was also decreased (Fig. 4*F*). We then examined the effect of PKCα siRNA on lyso-PAFAT activity by measuring the incorporation of acetyl-CoA into deuterium-labeled PAF. Knockdown of PKCα resulted in a significant decrease of lyso-PAFAT activation induced by mcPAF (Fig. 4*G*). From these data, we concluded that PKCα mediated the Ser-34 phosphorylation of LPCAT2 following mcPAFstimulation.

##### Activation and Phosphorylation of LPCAT2 by ATP Stimulation in RAW264.7 Cells Stably Expressing LPCAT2

**FIGURE 5.**
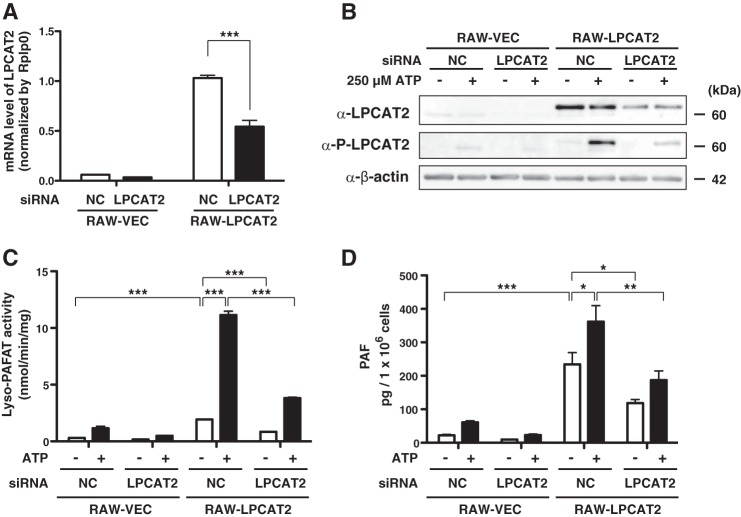
**LPCAT2 is the responsible enzyme for rapid PAF production.** RAW264.7 cells stably expressing vector (*RAW-VEC*) and FLAG-LPCAT2 (*RAW-LPCAT2*) were transfected with LPCAT2 siRNA by electroporation and incubated for 48 h. The cells were subsequently stimulated with 250 μm ATP for 30 s (*B* and *C*) or 1 min *(D). A*, the amount of mRNA of LPCAT2 was decreased by siRNA. *NC*, negative control. *B*, Western blot analysis showed decreased expression of both endogenous and FLAG-LPCAT2 by LPCAT2 siRNA. Signals from phospho-LPCAT2 were also reduced. *C*, lyso-PAFAT activity was measured by incorporation of acetyl-CoA into deuterium-labeled PAF. LPCAT2 siRNA reduced lyso-PAFAT activity in both RAW-VEC and RAW-LPCAT2. *D*, endogenous PAF production from each cellular extract was quantified using LC-MS/MS. Reduction of LPCAT2 protein expression by the siRNA was associated with decreased PAF production following ATP stimulation. Results are expressed as the mean + S.E. of three experiments (*A* and *D*) or mean + S.D. of an experiment performed in triplicate (*C*). *, *p* < 0.05; **, *p* < 0.01; ***, *p* < 0.001; analysis of variance and Tukey's multiple comparison test. The results (*C*) are representative of three independent experiments.

In addition to mcPAF, we investigated the ability for other G protein-coupled receptor ligands to mediate phosphorylation of LPCAT2 in RAW264.7 cells. Treatment with ATP led to phosphorylation of endogenous LPCAT2 within 30 s (Fig. 5). To confirm that LPCAT2 mediates PAF production in these cells, LPCAT2 was knocked down in RAW264.7 cells stably expressing either vector (RAW-VEC) or LPCAT2 (RAW-LPCAT2) (16). LPCAT2 mRNA and protein were decreased 48 h after transfection of LPCAT2 siRNA (Fig. 5, *A* and *B*). Endogenous expression of LPCAT2 in RAW264.7 cells was much lower than FLAG-LPCAT2. Phosphorylation of LPCAT2 following ATP stimulation was diminished correspondingly in cells transfected with LPCAT2 siRNA (Fig. 5*A*). Lyso-PAFAT activity in cellular lysates was measured by incorporation of acetyl-CoA into deuterium-labeled PAF. RAW-LPCAT2 cells had higher basal lyso-PAFAT activity than RAW-VEC cells, and ATP-stimulation increased the activity (Fig. 5*C*). When LPCAT2 was knocked down, both RAW-VEC and RAW-LPCAT2 showed lower lyso-PAFAT activity (Fig. 5*C*).

PAF production from RAW-VEC and RAW-LPCAT2 after 1 min of ATP stimulation was quantified by LC-MS/MS. The amount of PAF was elevated in RAW-LPCAT2 and increased upon ATP stimulation for 1 min. Knockdown of LPCAT2 by siRNA led to decreased LPCAT2 phosphorylation and PAF production (Fig. 5, *B* and *D*), compatible with lyso-PAFAT activity *in vitro* (Fig. 5*C*). From these data, we conclude that LPCAT2 is the enzyme that catalyzes the rapid PAF production following ATP stimulation.

##### PKCα Regulated the Phosphorylation of LPCAT2 following ATP Stimulation in RAW 264.7 Cells

**FIGURE 6.**
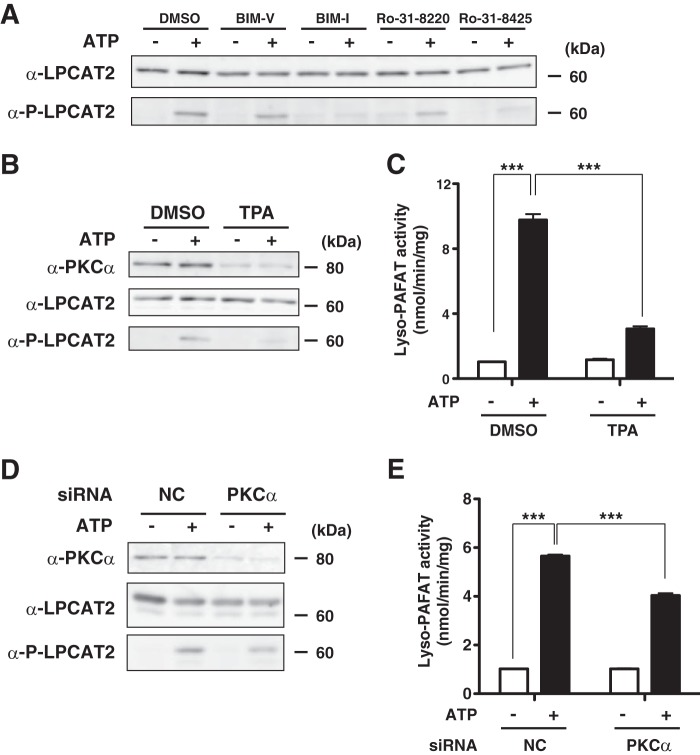
**PKCα mediates the phosphorylation of LPCAT2 following ATP stimulation.** *A*, non-transfected RAW264.7 cells were pretreated with 10 μm BIM-I, Ro-31-8220, Ro-31-8425, or BIM-V, an inactive analog of BIM-I, and stimulated with 250 μm ATP for 30 s. The cellular proteins were analyzed by Western blot analysis. Pretreatment with BIM-I, Ro-31-8220, or Ro-31-8425, but not BIM-V, abolished LPCAT2 phosphorylation. *DMSO*, dimethyl sulfoxide. *B*, RAW-LPCAT2 cells were treated with 100 nm TPA for 24 h and subsequently stimulated with ATP. *C*, lyso-PAFAT activity was measured by incorporation of acetyl-CoA into deuterium-labeled PAF. Pretreatment with TPA decreased the activation of lyso-PAFAT activity. *D*, the cellular proteins were analyzed by Western blot analysis. siRNA-mediated knockdown of PKCα protein was associated with decreased phosphorylation of LPCAT2 following ATP-stimulation. *NC*, negative control. *E*, decreased PKCα expression resulted in reduced lyso-PAFAT activation following ATP stimulation. Results are expressed as the mean + S.D. of an experiment performed in triplicate. ***, *p* < 0.001; analysis of variance and Tukey's multiple comparison test. The results are representative of three independent experiments with similar results.

To examine whether the PKC pathway also mediates ATP-induced LPCAT2 phosphorylation, non-transfected RAW264.7 cells were pretreated with PKC inhibitors and subsequently stimulated with 250 μm ATP for 30 s. The proteins were analyzed by Western blot analysis (Fig. 6*A*). Similar to the experiments using CHO-S-PAFR cells (Fig. 4), the inhibitors diminished phosphorylation of LPCAT2, which indicated that mcPAF and ATP utilize the same signaling pathway.

To down-regulate cPKC, RAW-LPCAT2 cells were treated with 100 nm TPA for 24 h and subsequently stimulated with 250 μm ATP for 30 s. Prolonged exposure to TPA treatment decreased the amount of PKCα protein (Fig. 6*B*). The phosphorylation of LPCAT2 by ATP stimulation was also reduced, accompanied by decrease in lyso-PAFAT activity *in vitro* (Fig. 6*C*). These two pharmacological experiments showed that PKCα mediated Ser-34 phosphorylation of LPCAT2.

We targeted PKCα by siRNA to examine the involvement of this isoform. Forty-eight hours after transfection with PKCα siRNA, the cells were treated with ATP for 30 s. PKCα protein was decreased by transfection of PKCα siRNA (Fig. 6*D*). Knockdown of PKCα led to reduced phosphorylation of LPCAT2 after ATP stimulation. However, some residual phosphorylation was still detected, possibly because of residual PKCα protein still present in the cell because of incomplete knockdown (Fig. 6*D*).

We then examined the lyso-PAFAT activity of LPCAT2 by measuring incorporation of acetyl-CoA into deuterium-labeled PAF. Knockdown of PKCα resulted in significant decrease of lyso-PAFAT activation after ATP stimulation (Fig. 6*E*). These data demonstrate that PKCα mediates the phosphorylation of LPCAT2 following ATP stimulation. However, a partial redundancy of other kinases cannot be fully excluded.

## DISCUSSION

This study establishes that rapid PAF biosynthesis in response to mcPAF or ATP stimulation requires PKCα-dependent Ser-34 phosphorylation of LPCAT2 (Figs. 4 and 6), whereas phosphorylation of the same residue following 30 min of LPS stimulation is mediated by MK2, as reported previously (16).

Our results from stable expression and knockdown of LPCAT2 confirmed that this enzyme is responsible for rapid PAF production (Fig. 5). Our experiments using PKC inhibitors and TPA showed that activation of cPKC is essential for the Ser-34 phosphorylation of LPCAT2 by mcPAF or ATP (Figs. 4 and 6). Among the three PKC subfamilies (39), only cPKC requires Ca^2+^ for its activation, whereas neither novel PKC nor atypical PKC exhibit Ca^2+^ dependence. Our knockdown experiments established the positive involvement of PKCα on Ser-34 phosphorylation. However, a partial redundancy of other isoforms or kinases cannot be fully excluded in this study (Figs. 4 and 6).

Previously, we demonstrated that PAF production by LPCAT2 is up-regulated following LPS stimulation by two modes: phosphorylation of LPCAT2 at Ser-34 by MK2 occurs within 30 min (16), and LPCAT2 mRNA is increased by 16 h (9). LPS is a major component of the outer membrane of Gram-negative bacteria, and PAF production in response to bacterial infection likely functions in the proinflammatory reaction of host defense. Here, we established a new third pathway and mechanism to enhance PAF biosynthesis following stimulation by endogenous G protein-coupled receptor ligands within a shorter time period (30 s). It is possible that G protein-coupled receptor signaling works as the direct trigger for PAF production, whereas LPS-stimulation serves as preconditioning for the subsequent inflammatory state. Besides its many intracellular and intercellular functions, ATP functions as a damage-associated molecular pattern molecule and modulates immune responses following release from the cell during cellular stress or tissue injury (43). The novel pathway described here suggests roles of PAF during intrinsic events such as necrosis, apoptosis, and tissue damage by ischemia-reperfusion (44). Because PAF works as a chemoattractant (30), we hypothesize that rapid production of PAF by phosphorylated LPCAT2 in tissue macrophages recruits more immune and stromal cells to the lesion to repair damaged tissue (20). Because LPCAT2 was activated by its own product, PAF, a positive feedback mechanism for PAF production that may function at the onset of inflammation is indicated.

The regulation of LPCAT2 in macrophages implies stage-specific roles of PAF in a wide variety of proinflammatory responses from the innate to the adaptive immune systems. Rapid production of PAF by phosphorylated LPCAT2 may trigger inflammatory reactions such as increased blood vessel permeability or chemical mediator release at the onset of infections or type I allergy (30). PAF-stimulated LPCAT2 phosphorylation and endogenous PAF production decrease rapidly when activated *in vitro* (Fig. 2, *B* and *C*), suggesting that this role of PAF is required for a very short period, consistent with the rapid degradation of PAF by PAF acetyl hydrolase in the cytosol and plasma (10–12). Production and degradation of PAF seem strictly controlled within a limited area and time span, indicating that PAF may function as a trigger for subsequent events during pathophysiological circumstances.

On the other hand, the LPCAT activity of LPCAT2 was also increased during the same rapid time course (Fig. 3*C*). LPCAT2 selectively catalyzes the incorporation of polyunsaturated fatty acids, such as arachiconic acid, into 1-alkyl-PC at the *sn*-2 position. 1-alkyl-PC containing arachidonic acid is a major membrane phospholipid of inflammatory cells (14). The physiological roles of 1-alkyl-PC production by LPCAT2 remain to be elucidated, but it possibly regulates the properties of membrane phospholipids in response to the inflammatory stimuli. It is unclear whether incorporation of arachidonic acid into the cellular membranes either increases or decreases its bioavailability for subsequent eicosanoid production.

The data obtained in this study reveal a novel mechanism for PAF production that may function at the onset or early stages of inflammation. Because LPCAT2 may be a key enzyme balancing pro- and anti-inflammatory lipid metabolism, additional studies are necessary to understand its functions at various stages of inflammation, which may elucidate potential targets for medical intervention. For example, selective inhibition of the lyso-PAFAT activity of phosphorylated LPCAT2 might ameliorate excessive inflammation in response to exogenous or endogenous triggers. Thus, further *in vivo* studies are required to elucidate the roles of phosphorylated LPCAT2 under inflammatory conditions.

## Footnotes

3 The abbreviations used are:

PAF: platelet-activating factor
PAFR: platelet-activating factor receptor
1-alkyl-PC: 1-*O*-alkyl-2-acyl-*sn*-glycero-3-phosphocholine
lyso-PAF: lyso-PAF, 1-*O*-alkyl-*sn*-glycero-3-phosphocholine
LPC: lysophosphatidylcholine
lyso-PAFAT: acetyl-CoA:1-*O*-alkyl-*sn*-glycero-3-phosphocholine acetyltransferase
LPCAT: lysophosphatidylcholine acyltransferase
PC: phosphatidylcholine
mcPAF: methylcarbamyl platelet-activating factor
BIM: bisindolylmaleimide
TPA: 12-*O*-tetradecanoylphorbol-13-acetate
cPKC: conventional PKC
MS/MS: tandem mass spectrometry.
